# Allosteric modulation of protein oligomerization: an emerging approach to drug design

**DOI:** 10.3389/fchem.2014.00009

**Published:** 2014-03-24

**Authors:** Ronen Gabizon, Assaf Friedler

**Affiliations:** Institute of Chemistry, The Hebrew University of JerusalemJerusalem, Israel

**Keywords:** protein oligomerization, peptides, HIV-1, drug design, allostery, shiftides

## Abstract

Many disease-related proteins are in equilibrium between different oligomeric forms. The regulation of this equilibrium plays a central role in maintaining the activity of these proteins *in vitro* and *in vivo*. Modulation of the oligomerization equilibrium of proteins by molecules that bind preferentially to a specific oligomeric state is emerging as a potential therapeutic strategy that can be applied to many biological systems such as cancer and viral infections. The target proteins for such compounds are diverse in structure and sequence, and may require different approaches for shifting their oligomerization equilibrium. The discovery of such oligomerization-modulating compounds is thus achieved based on existing structural knowledge about the specific target proteins, as well as on their interactions with partner proteins or with ligands. *In silico* design and combinatorial tools such as peptide arrays and phage display are also used for discovering compounds that modulate protein oligomerization. The current review highlights some of the recent developments in the design of compounds aimed at modulating the oligomerization equilibrium of proteins, including the “shiftides” approach developed in our lab.

## Introduction

Oligomerization is a common property of proteins and takes place in all biological systems. It is estimated that at least 35% of all proteins in cells are oligomeric (Jones and Thornton, 1996; Goodsell and Olson, 2000). The properties of protein oligomers are highly diverse: Protein oligomers may be homoligomers (Wang et al., 1994; Eisenstein and Beckett, 1999; Yun et al., 2007; Thulin et al., 2011) or heterooligomers (Fermi et al., 1984; Cramer et al., 2001; Unwin et al., 2002; Gomez et al., 2011) and can range from dimers (Sapienza et al., 2007) to high order structures such as capsids (Wu and Rossmann, 1993; Nam et al., 2011) and fibrils (Craig and Woodhead, 2006; Bedrood et al., 2012). Some proteins form one specific active oligomeric state. This is the case with hemoglobin, which exists in red blood cells as an α_2_β_2_ heterotetramer (Fermi et al., 1984), and with the acetylcholine receptor that is a pentamer (Unwin et al., 2002). Other proteins are involved in dynamic oligomerization equilibria between several states with different activities, and switch between these states as part of regulating their normal function. For example, the enzyme carbamoyl phosphate synthase (CPS) exists in equilibrium between inactive dimers and active tetramers. The equilibrium is regulated by allosteric inhibitors that stabilize the dimer and allosteric activators that stabilize the tetramer (Kim and Raushel, 2001; Mora et al., 2002). In many cases, the dynamic assembly and disassembly of oligomers plays a central role in regulating the activity of a protein, as in the case of actin (Ono, 2007), which induces cell motility by this mechanism.

Correct protein oligomerization is critical for its function and is therefore tightly regulated by various factors. For example, the GroEL-GroES chaperonin complex assists in the folding of polypeptides under thermal stress conditions. The complex undergoes small changes in the inter-subunit interactions when the temperature increases from 37 to 42–45°C. This may enable it to distinguish normal temperatures from stress temperatures (Cabo-Bilbao et al., 2006). Protein oligomerization can also be regulated by the binding of partner proteins (Grossman, 2001; Fernandez-Fernandez et al., 2005; Marinho-Carvalho et al., 2006; De Meyts, 2008; Rajagopalan et al., 2008), metal cofactors or small molecule allosteric effectors (Kim and Raushel, 2001; Lawrence et al., 2008; Selwood et al., 2008; Semenova and Chernoff, 2012). In a notable example, Krojer et al. discovered that the bacterial HtrA protease exists in an inactive hexameric state, which undergoes an extensive conformational change upon binding to effector peptides. This change involves the rearrangement of the active site to a catalytically active structure and induces the formation of 12-mer or 24-mer oligomeric states (Krojer et al., 2010). Changes in the strength and geometry of interactions between subunits can also be modulated by ATP hydrolysis (Zhang et al., 2010).

Since oligomerization is very common and is crucial for protein activity, modulating this process is a highly promising therapeutic strategy that can be applied to many different diseases involving oligomeric proteins (Hayouka et al., 2007; Lawrence et al., 2008; Christ et al., 2012; Gabizon et al., 2012). Several compounds that are already in clinical use were later discovered to act via modulation of protein oligomerization. A well-known example is that of the anti-cancer drug Taxol, which was discovered in the stem bark of the pacific yew during the screening of plant-derived compounds for cytotoxic activity (Wani et al., 1971). It was later discovered that Taxol binds to β-tubulin (Löwe et al., 2001) and allosterically inhibits the assembly and disassembly dynamics of microtubules (Jordan et al., 1993; Derry et al., 1995), thus interfering with mitosis and inhibiting the division of cancer cells.

Many compounds that modulate protein oligomerization are still being discovered indirectly by screening methods that do not target protein oligomerization. However, a growing number of studies aim to discover molecules that directly target the oligomerization of a well characterized protein. The understanding of oligomeric protein structures and how they are regulated significantly progressed in recent years. Structures determined by NMR and X-ray crystallography enable detailed characterization of the oligomerization interfaces and the interactions that stabilize the oligomers (see for example Fermi et al., 1984; Jeffrey et al., 1995; Lange-Savage et al., 1997; Luger et al., 1997; Walters et al., 1997; Whitson et al., 2005; Sharma et al., 2010). Methods for precise determination of the oligomeric states of a protein and their relative populations, such as size exclusion chromatography (SEC) (Mateu and Fersht, 1998; Gotte et al., 2012; Yu et al., 2013), analytical ultracentrifugation (AUC) (Weinberg et al., 2004a; Murugan and Hung, 2012; Szymanski et al., 2013) and single molecule methods (Groulx et al., 2011; Paredes et al., 2012; Calebiro et al., 2013), improve our understanding of the thermodynamics and kinetics of oligomerization processes. Based on this knowledge, molecules that shift the oligomerization equilibrium of target proteins are being developed using techniques ranging from de novo design to combinatorial screening. This review will cover the latest developments in this field and their application to various disease-related proteins. The compounds discussed in this study are summarized in table S1.

## Mechanisms of modulating the oligomerization equilibrium of proteins

**Figure 1:**
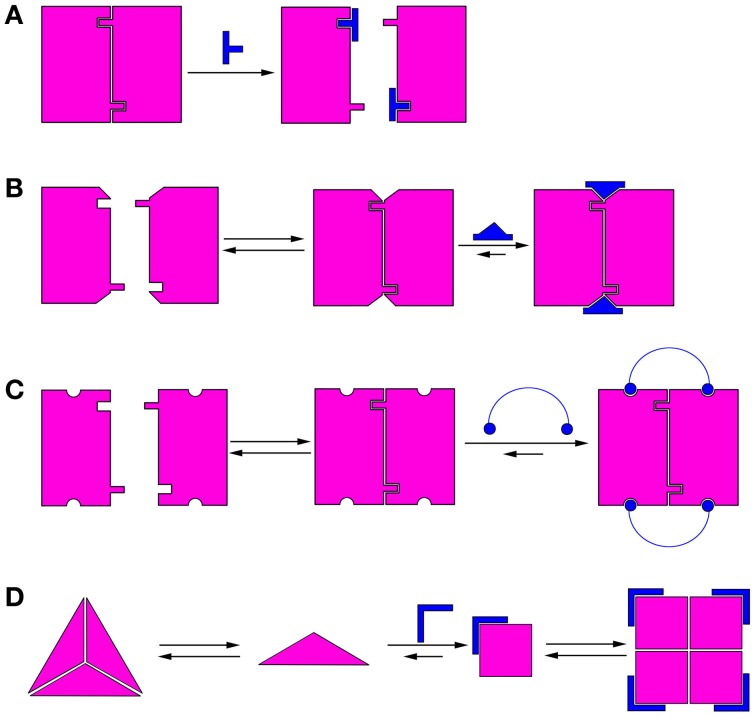
**Schematic illustrations of mechanisms of modulation of protein oligomerization. (A)** Direct blocking of oligomerization interfaces; **(B)** Binding to or near the oligomerization interface and stabilizing it; **(C)** Binding to several monomers simultaneously; **(D)** Morpheein mechanism (Jaffe, 2005). The conformations determine the stoichiometry of the full oligomer. In this example, a ligand binds the monomer and stabilizes the conformation that promotes formation of a different oligomer.

Modulation of protein oligomerization can take place by various mechanisms. In the simplest mechanism, inhibition of oligomerization can be achieved by molecules that bind directly to the oligomerization interface and competitively block it (He et al., 2005), thus preventing oligomerization (Figure 1A). These competitive oligomerization inhibitors do not act by stabilizing a specific oligomeric state and are thus outside the scope of this review. Alternatively, molecules may stabilize a specific oligomer by binding near the oligomerization interface (Kessl et al., 2009) (Figure 1B) or by binding to several monomers simultaneously (Teufel et al., 2007) (Figure 1C).

A different mechanism for allosteric modulation of oligomerization equilibria was described by Jaffe et al. (Jaffe, 2005). In this mechanism, a lower oligomeric form of the protein can exist in different conformations (called morpheeins), and each conformation dictates a defined stoichiometry for the higher oligomer. Transition between the oligomeric states requires dissociation of the oligomer and a change in conformation before the other oligomeric state is formed. Therefore, molecules that allosterically stabilize a certain conformation of the lower oligomer will shift the oligomerization equilibrium toward the corresponding higher oligomer (Figure 1D). This mechanism has been shown for the enzyme phorphobilinogen synthase (PBGS), which is involved in tetrapyrrole metabolism and plays a crucial role in cellular respiration (Jaffe and Lawrence, 2012). PBGS exists in equilibrium between active octamers and inactive hexamers. The transition between the two states requires dissociation into dimers followed by a conformational change in the dimer and reassociation. This process can be modulated by allosteric effectors, such as magnesium, which specifically stabilizes the octamer (Jaffe, 2005). Lawrence et al. used *in silico* screening to develop a compound that inhibits pea PGBS by binding specifically to the inactive hexamer (Lawrence et al., 2008), thus proving the feasibility of modulating the activity of proteins by shifting their oligomerization equilibrium. Many proteins exhibit characteristics indicating that their oligomerization dynamics follow the morpheein mechanism (reviewed in Selwood and Jaffe, 2012).

## The tumor suppressor p53

The tumor suppressor p53 is a transcription factor that is activated and accumulated in the nucleus in response to oncogenic stress. Following its induction, p53 binds specific promoters in the genome and activates the transcription of a wide array of target genes, aimed at eliminating the threat of malignant transformation (Levine, 1997; Vogelstein et al., 2000; Ryan et al., 2001; Michael and Oren, 2002). p53 is mutated in over 50% of all cancer cases, highlighting the vital role it plays in tumor suppression. The majority of cancer-associated mutations in p53 occur in its DNA binding core domain (Levine, 1997).

p53 is active as a homotetramer (Chene, 2001) and its tetramerization is mediated by a structurally independent tetramerization domain (p53Tet, residues 326–355) (Clore et al., 1994; Lee et al., 1994; Jeffrey et al., 1995). Tetramerization of p53 is vital to its function and plays a central role in the regulation of p53 activity. p53 tetramers bind p53 DNA response elements more tightly than dimers and monomers, and only tetramers can induce transcription of p53 target genes (Weinberg et al., 2004b; Menendez et al., 2009). Tetramerization also affects the cellular localization of p53: the Nuclear Export Signal (NES) of p53 is located within the tetramerization domain and is shielded in p53 tetramers, preventing nuclear export of p53 tetramers (Stommel et al., 1999). However, in monomers and dimers of p53 the NES is exposed and p53 is thus exported from the nucleus to the cytoplasm, where it is degraded via the ubiquitin-proteasome pathway.

The oligomerization equilibrium of p53 is regulated by interactions with other proteins, such as proteins from the 14-3-3 and S100 families (Fernandez-Fernandez et al., 2005, 2008; Rajagopalan et al., 2008; Słomnicki et al., 2009; Van Dieck et al., 2009a) and numerous kinases (Delphin et al., 1997; Gotz et al., 1999). Post translational modifications also have an effect on p53 oligomerization, either by directly affecting tetramer stability (Nomura et al., 2009; Yakovlev et al., 2010) or by modulating the interactions of p53 with other proteins (Rajagopalan et al., 2008; Van Dieck et al., 2009b). Recently, using fluorescence correlation spectroscopy in single cells, Gaglia et al. showed that DNA damage causes the shifting of the oligomerization equilibrium of p53 toward tetramers, and that this change is sufficient to activate the transcription of p53 target genes even without the net accumulation of p53 (Gaglia et al., 2013). The importance of tetramerization for p53 function makes p53 an attractive therapeutic target for compounds that modulate protein oligomerization. Several recent projects utilized different strategies to shift the oligomerization equilibrium of p53 toward the active tetramer.

**Figure 2:**
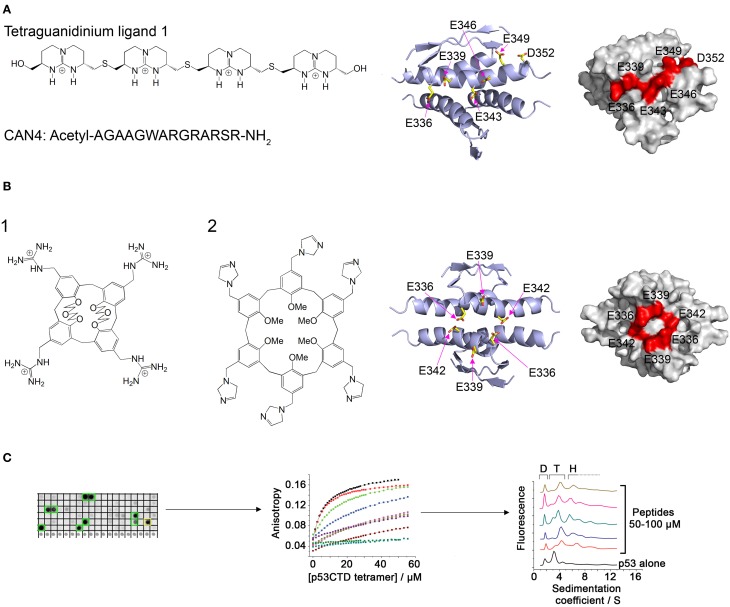
**Modulation of p53 oligomerization. (A)** Left: Structure of the tetraguanidinium ligand described in Salvatella et al. (2004) and sequence of the peptide CAN4 described in Martinell et al. (2006). Right: Stick model and space filling model (indicated residues in red) of the acidic patch on the surface of the p53 tetramerization domain targeted by the two molecules. Structures from pdb 1PET (Lee et al., 1994); **(B)** Left: Structure of guanidinium-calix[4]arene described in Gordo et al. (2008) (1) and imidazole calix[6]arene described in Kamada et al. (2010) (2). Right: structure of the acidic patch targeted by these molecules; **(C)** Discovery of peptides that bind specifically to tetrameric p53. A peptide array derived from p53-binding proteins was screened for binding to p53CTD (left). The binding of the identified peptides was quantified by fluorescence anisotropy (center) and their effect on p53 oligomerization was characterized using AUC (right).

Ligands containing several spaced cationic groups bound the p53 tetramerization domain and stabilized p53 tetramers. These ligands were developed using a combination of intuitive design with computational and combinatorial methods. Salvatella et al. designed a tetraguanidinium ligand that binds to a patch of negatively charged residues on the surface of the p53 tetramerization domain (Figure 2A), facing outwards from the dimer-dimer interface (Salvatella et al., 2004). This ligand was used by Martinell et al. as a basis for the computational design of a peptide with four arginine residues with similar spacing as the guanidinium groups in the original ligand. The new peptide (CAN4) bound p53Tet with a *K*_*d*_ of 8 μM and increased the thermal stability of p53Tet by 2°C. The same group later synthesized a library of modified peptides and tested their binding to p53Tet. Several peptides in the library bound p53Tet with affinities as low as 0.8 μM (Martinell et al., 2006).

Gordo et al. focused on the cancer associated R337H mutant of p53, in which a critical hydrogen bond is lost due to the mutation, resulting in destabilization of the p53 tetramer. They designed a calix[4]arene with 4 guanidiniomethyl groups, in which a hydrophobic calixarene group binds to the hydrophobic pocket formed in the dimer-dimer interface of the p53Tet tetramer and the guanidinium groups form hydrogen bonds with acidic residues located on different monomers (Figure 2B). The designed ligand bound the R337H mutant and increased the thermal stability of the mutant to the same level as the wild type p53Tet (Gordo et al., 2008). In a later work, Gordo et al. showed that increasing the flexibility of the ligand increases its affinity to the R337H by enabling it to accommodate the optimal geometry for binding more easily (Gordo et al., 2011). However, the activity of this ligand was tested in water, and experiments at physiological ionic strength did not show any activity (Kamada et al., 2010). Based on this work, Kamada et al. Designed larger ligands with a calix[6]arene group with different positively charged end groups. One of the ligands, containing imidazole groups, increased the thermal stability of the R337H and enhanced the transcriptional activity of p53 R337H in cells (Kamada et al., 2010).

A different approach to discovering molecules that stabilize p53 tetramers was used in our laboratory (Gabizon et al., 2008, 2012). We used the natural protein-protein interactions of the p53 tetramerization domain or nearby regions for developing peptides that bind p53 in or near its tetramerization domain and may thus stabilize the tetramer. The interaction between p53Tet and the HIV-1 Tat protein (Longo et al., 1995) was characterized using peptide mapping. Two peptides from HIV-1 Tat bound p53Tet, and the interaction of a peptide derived from the arginine rich motif of Tat with p53Tet was characterized. The Tat-derived peptide bound all oligomeric forms of p53Tet without preference and thus was not a potential candidate for modulating p53Tet oligomerization (Gabizon et al., 2008). In a following work, we used a combinatorial approach and designed a peptide array derived from proteins known to bind the C terminal domain (CTD) of p53 (p53CTD, residues 293–393). Screening the array with recombinant p53CTD resulted in the identification of 10 peptides that bound p53CTD. Several of these peptides increased the thermal stability of the p53CTD and bound specifically to p53 tetramers in AUC experiments (Gabizon et al., 2012) (Figure 2C).

Other cancer related proteins may also be potential targets for molecules that modulate their oligomerization. Gray et al. developed a fluorescent monoclonal antibody assay for determining the extent of oligomerization of the oncoprotein AGR2 (Gray et al., 2013). They found that a peptide derived from the disordered N-terminal domain (NTD) of AGR2 stabilizes AGR2 oligomers, and that oligomerization of AGR2 enhances its binding to its chief partner protein reptin. This highlights the therapeutic potential of compounds that modulate AGR2 oligomerization.

**Figure 3:**
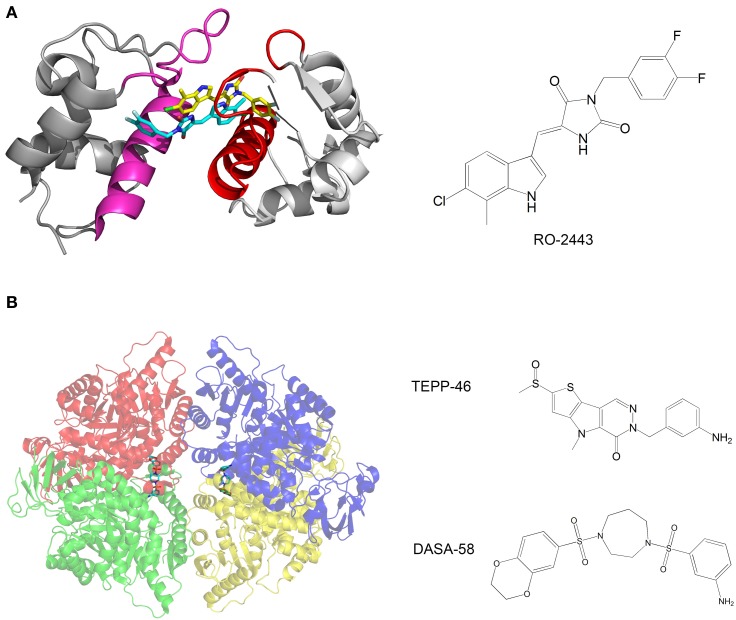
**(A)** Crystal structure of mdmX with the inhibitor RO-2443. A dimer of RO-2443 (sticks, yellow, and cyan) induces dimerization of mdmX (each monomer in different shade of gray). The binding sites for p53 (red and magenta) are blocked in the dimer. Structure from pdb 3U15 (Graves et al., 2012); **(B)** Crystal structure of pyruvate kinase M2 (PKM2) with the activator DASA-58. The four monomers are in red, green, blue and yellow and are semitransparent. DASA-58 is in cyan. The second activator TEPP-46 binds at the same site. Structure from pdb 3ME3 (Anastasiou et al., 2012).

Mdm2 and mdmX are negative regulators of p53 that mediate polyubiquitination and degradation of p53. Graves et al. developed compounds that bind mdm2 and mdmX and induce dimerization of the proteins. The p53-binding interface is buried in the dimerization interface, thus inhibiting the binding of p53 by mdm2 and mdmX (Figure 3A). The compounds activate the p53 transcriptional pathway in cells and induce apoptosis of cancer cells (Graves et al., 2012).

Another example of anti-cancer compounds that act by promoting protein oligomerization is given by the work of Anastasiou et al. (2012). In cancer cells, inhibition of the enzyme pyruvate kinase M2 (PKM2) by phosphotyrosine-containing proteins increases the availability of glycolytic metabolites for the support of cell proliferation. The authors characterized two small molecule activators of PKM2. The compounds activate PKM2 by stabilizing its tetrameric state, prevent inhibition of PKM2 by phosphotyrosine-containing protein, alter the metabolism of cancer cells and inhibit cancer cell proliferation. Structural studies of the compounds with PKM2 revealed that the compounds bind at the interface between two monomers and stabilize the tetrameric structure (Figure 3B).

Another potential target may be BAK, a mitochondrial membrane protein involved in apoptosis. Following cell stress, p53 binds to BAK and induces BAK oligomerization (Leu et al., 2004; Pietsch et al., 2008), a critical stage in mitochondrial apoptosis. Thus molecules that bind and induce oligomerization of BAK may have potential as anti-cancer drug leads.

## Viral proteins

Oligomeric proteins play vital roles in the replication cycles of viruses. Many viruses encode proteases that catalyze the processing of viral polyproteins or the maturational processing of precapsids. These proteases are frequently oligomeric, as in HIV (Lange-Savage et al., 1997) and hepatitis C virus (Li et al., 2010). The function of viral capsid proteins is derived from their oligomerization properties, and the high order structures of numerous capsids have been characterized, as in the case of canine parvovirus (Wu and Rossmann, 1993), Hepatitis B virus (HBV) (Katen et al., 2013), and HIV (Zhao et al., 2013). Other prominent viral proteins that are active as oligomers include integrases (Cherepanov et al., 2003) and reverse transcriptases (Smerdon et al., 1994). Targeting the oligomerization of these proteins is emerging as a promising therapeutic strategy.

Considerable work has been performed on directly inhibiting the oligomerization of viral proteins using molecules that bind to their oligomerization interfaces. Peptides derived from the oligomerization interfaces can inhibit the oligomerization and activity of such proteins, as was shown for the HIV-1 reverse transcriptase (Divita et al., 1994; Morris et al., 1999; Depollier et al., 2005) and integrase (Sourgen et al., 1996; Maroun et al., 2001; Zhao et al., 2003). Non peptidic inhibitors of protein oligomerization have also been characterized (Rodríguez-Barrios et al., 2001; Bonache et al., 2005; Koh et al., 2007; Vidu et al., 2010; Tintori et al., 2012). This subject has been reviewed elsewhere and is outside the scope of the current review (Camarasa et al., 2006).

Inhibition of viral proteins can also be achieved by ligands that do not bind the oligomerization interface, but rather modulate the oligomerization of the protein allosterically. One of the main targets for this approach is the HIV-1 integrase protein (IN), which catalyzes the integration of the viral cDNA into the host genome (Delelis et al., 2008), a crucial step in the HIV-1 replication cycle (Sherman and Greene, 2002). Integration proceeds by two steps, each performed by a specific IN oligomer: (i) 3′ end processing, in which IN removes a GT dinucleotide from the 3′ termini of the long terminal repeats (LTRs) in the viral DNA. This step is performed by IN dimers (Deprez et al., 2001; Guiot et al., 2006) bound to each LTR in the cytoplasm; (ii) strand transfer, in which the viral DNA is integrated into the host DNA following nuclear import of the stable synaptic complex (Engelman et al., 1991). This step is performed by tetrameric IN (Li and Craigie, 2005; Li et al., 2006) in the nucleus with the assistance of cellular proteins, especially LEDGF/p75, which promotes IN tetramerization and tethers it to the chromosomes (Cherepanov et al., 2003; Maertens et al., 2003; Emiliani et al., 2005). The protein-protein interactions of IN are emerging as promising therapeutic targets (Maes et al., 2012), in particular the IN-LEDGF/p75 interaction (Christ and Debyser, 2013).

The importance of the integration step within the HIV replication cycle and the lack of mammalian homologs for IN both make IN an attractive therapeutic target. However, only two IN inhibitors (Raltegravir and Elvitegravir) are currently used in the clinic as anti-HIV drugs (Serrao et al., 2009; Messiaen et al., 2013). The rapid development of resistant strains (Mouscadet et al., 2010) emphasize the need to develop new drugs that function by different mechanisms, and efforts are made by numerous laboratories to discover novel IN inhibitors. In most cases, the modulation of IN oligomerization was not the intended result and candidate compounds were screened for inhibition of IN catalytic activity or inhibition of IN-LEDGF/p75 binding. However, many of the most promising IN inhibitors discovered recently were later shown to act primarily by shifting the oligomerization equilibrium of IN.

**Figure 4:**
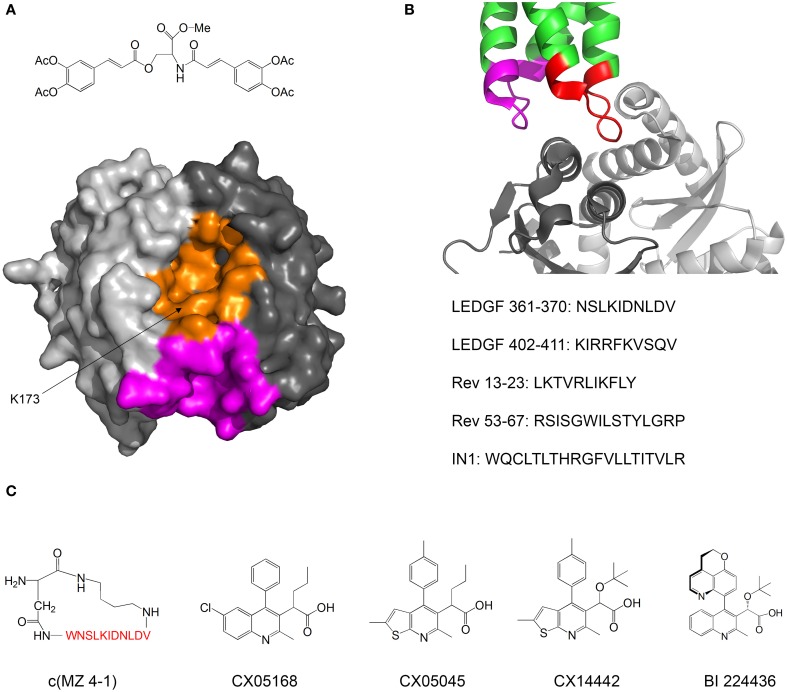
**Inhibiting of HIV-1 IN by modulating its oligomerization equilibrium. (A)** Structure of the IN inhibitor described by Kessl et al. (2009) and Possible binding sites. The two monomers of the IN CCD are in two shades of gray. The possible binding sites for the inhibitor are in orange and magenta. Structure from pdb 1EXQ (Chen et al., 2000). **(B)** Structure of the complex between the IN CCD (dimer, two shades of gray) and the IN binding domain of LEDGF/p75 (green). The LEDGF sequences that mediate IN binding are residues 361–370 (red) and 402–411 (magenta). The sequences of peptide inhibitors derived from LEDGF (Hayouka et al., 2007), Rev (Hayouka et al., 2008) and from combinatorial screening (Maes et al., 2009) are given. Structure from pdb 2B4J (Cherepanov et al., 2005); **(C)** Structures of IN inhibitors described in the review (from left to right): c(MZ 4-1), a cyclic derivative of LEDGF 361–370 (Hayouka et al., 2010b). Amino acid residues are shown in red; Inhibitors CX05168, CX05045, and CX014442 Described by Christ et al. (2010, 2012); inhibitor BI 224436 (Fader et al., in press).

Kessl et al. studied IN inhibitors derived from chicoric acid, which inhibit the strand transfer activity of IN at micromolar concentrations (Kessl et al., 2009). They developed a method to measure the rate of subunit exchange between IN oligomers. The method uses His_6_-labeled IN and non-labeled IN, which are allowed to equilibrate separately and then mixed. The exchange of subunits is monitored by pulling down the His_6_-IN with Ni^+2^ beads and measuring the amount of non-labeled IN pulled down by SDS PAGE. One of the compounds significantly reduced the extent of subunit exchange, indicating that the oligomers were stabilized and thus dissociated more slowly. Molecular docking and mutational analysis indicated that the compound binds at a cleft located at the interface between two monomers of the IN catalytic core domain (CCD), while forming contacts with both monomers, thus stimulating oligomerization (Figure 4A). The authors postulate that the compound induces the formation of conformationally rigid IN tetramers that are unable to bind DNA in the necessary orientation for catalysis.

In our laboratory we discovered peptides that bind IN and shift its oligomerization equilibrium toward the tetramer, thus inhibiting the 3′-end processing catalytic activity and preventing viral replication. We termed these peptides “Shiftides.” The first anti-IN shiftides were derived from the IN-binding site in the cellular protein LEDGF/p75 (Hayouka et al., 2007) (Figure 4B). These peptides bound IN, shifted its oligomerization equilibrium toward the tetramer and inhibited IN activity *in vitro* and in HIV-1 infected cells (Hayouka et al., 2010a). We then synthesized a series of cyclic peptides derived from the IN binding sites in LEDGF/75. One of the peptides inhibited IN as much as the linear peptide while being more stable in cells (Figure 4C) (Hayouka et al., 2010b). The mechanism of binding of the cyclic peptides to IN depended on their ring size—while peptides with smaller rings bound preferably to IN dimers and stabilized them, larger rings promoted binding and stabilization of IN tetramers (Hayouka et al., 2010b). A similar mechanism of inhibition was discovered for peptides derived from the viral protein Rev (Figure 4B) (Hayouka et al., 2008), which binds and inhibits IN (Rosenbluh et al., 2007). IN-inhibitory shiftides were also developed by a combinatorial approach: a 20 residue peptide termed IN1, which was identified by a yeast two hybrid assay, bound to tetrameric IN, shifted the oligomerization equilibrium of IN toward the tetramer and inhibited IN activity (Figure 4B) (Armon-Omer et al., 2008; Maes et al., 2009). In comparison, peptides comprising residues 1–10 or residues 11–20 of IN1 bound to IN dimers, indicating that IN1 may stabilize tetrameric IN by bridging two IN dimers (Maes et al., 2009).

A major class of novel IN inhibitors are termed Allosteric IN Inhibitors (ALLINI's) (Tsantrizos et al., 2007; Christ et al., 2012; Tsiang et al., 2012; Engelman et al., 2013; Fader et al., in press). Christ et al. used a combination of computational and experimental methods to discover IN-inhibiting compounds. The authors initially aimed to find inhibitors for the IN-LEDGF/p75 interaction and used virtual screening to design a library of compounds that may bind to the LEDGF/p75 binding sites in IN and inhibit the IN-LEDGF/p75 interaction. Several compounds with micromolar activities were discovered (Christ et al., 2010). Further optimization yielded the compound CX14442, which inhibited the IN-LEDGF/p75 interaction at submicromolar concentrations (Figure 4C) (Christ et al., 2012). The authors discovered that beyond inhibiting the IN-LEDGF/p75 interaction, the compounds enhance oligomerization of IN and directly inhibit IN activity. Kessl et al. further studied the mechanism of action of these compounds as well as a similar IN inhibitor discovered by high throughput screening for 3′ processing activity (Figure 4C) (Tsantrizos et al., 2007). The ALLINI's bound at the dimerization interface between two CCD monomers, promoted IN multimerization and increased the thermal stability of IN. Studies of an IN mutant which is resistant to inhibition by ALLINI's showed that inhibition of IN activity is achieved mainly by the promotion of IN multimerization and not by inhibition of the IN-LEDGF/p75 interaction (Feng et al., 2013). Furthermore, Jurado et al. found that ALLINI's promote IN multimerization in virions, and that virions produced in the presence of ALLINI's are not infectious and have no reverse transcriptase or integrase activity (Jurado et al., 2013). One of the compounds, BI 224436, has an EC50 of 11–27 nM and has recently entered clinical trials (Fader et al., in press). These studies highlight the versatility and potency of compounds that inhibit IN by modulating its oligomerization equilibrium.

Viral capsid proteins are also a promising target for compounds that modulate oligomerization. Viral capsids are large, highly symmetric oligomers comprised of one or several types of monomers, and may contain hundreds of subunits (Mateu, 2013). The function of capsid proteins requires that they form highly stable capsids that can withstand high internal pressures (Molineux and Panja, 2013) and yet be able to dissociate upon cell entry and release the viral DNA and proteins into the host cell. Incorrect formation of the viral capsid can be detrimental to the replication of viruses and thus mutations that destabilize or alter the structure of the capsid highly reduce the infectivity of viruses, as in the case of HIV (Noviello et al., 2011) and HBV (Tan et al., 2013). Therefore, many inhibitors targeted against capsid proteins are currently being developed.

**Figure 5:**
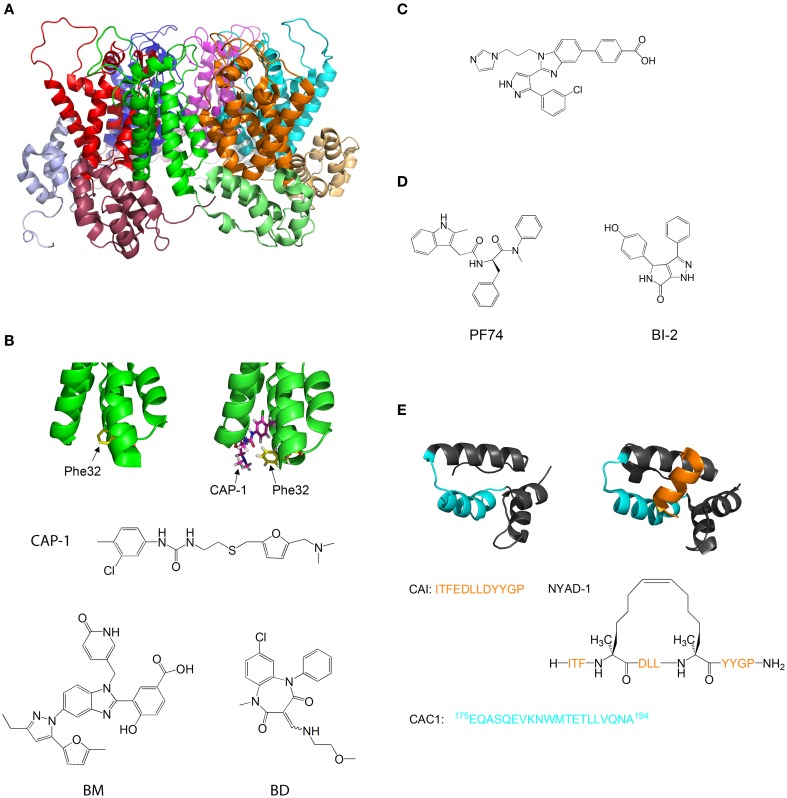
**Molecules that target capsid assembly. (A)** Structure of HIV-1 capsid protein hexamer. Different monomers are in Green, red, blue, magenta, cyan, and orange. The NTD and CTD of every monomer are in different shades of the same color. Structure from pdb 1VUU (Zhao et al., 2013). **(B)** Structure of HIV-1 capsid protein NTD in the absence (left) and presence (right) of CAP-1. Phe32 is shown in yellow, CAP-1 is shown in magenta. Structures from pdb files 1VUU (Zhao et al., 2013) and 2JPR (Kelly et al., 2007). The formulas of CAP-1 and a representative Benzimidazole (Goudreau et al., 2013) and Benzodiazepine (Tremblay et al., 2012) are given at the bottom. **(C)** Formula of CA-binding compound described by Lemke et al. (2013). **(D)** Formula of the CA-binding compounds PF74 (described by Shi et al., 2011) and BI-2 (described by Lamorte et al., 2013) **(E)** Structure of HIV-1 capsid protein CTD in the absence (left) and presence (right) of the capsid assembly inhibitor (CAI). CAI is in orange. The helix from which the peptide CAC1 was derived (Bocanegra et al., 2011) is in cyan. The sequence of CAI and the structure of the optimized peptide NYAD-1 (Zhang et al., 2008) are given below. Structures from pdb files 1AUM (Gamble et al., 1997) and 2BUO (Ternois et al., 2005).

The HIV-1 capsid protein (CA) is one of the main targets for inhibition. CA is composed of two helical domains, the NTD and the CTD, which are connected via a flexible linker. The basic structural unit of the capsid is a hexameric ring formed by NTD-NTD interactions, and reinforced by intermolecular NTD-CTD interactions (Figure 5A). Dimeric CTD-CTD interactions link between the hexameric rings, and the full capsid has a fullerene-like structure (Pornillos et al., 2009). Correct assembly of the capsid may be very sensitive to small changes in the strength and geometry of the inter-subunit interactions. Unlike in the case of HIV-1 integrase, most HIV inhibitors that target CA were intended for inhibition of capsid formation and were discovered by rational structure-based approaches or screenings that used *in vitro* capsid assembly assays.

Tang et al. used virtual screening to search for compounds that bind and inhibit CA. One of the compounds discovered, CAP-1, inhibits HIV-1 infectivity by specifically interfering with the formation of viral capsids (Tang et al., 2003). CAP-1 inhibited capsid formation *in vitro* and caused the formation of viral capsids with abnormal morphologies. ^1^H-^15^N HSQC measurements showed that CAP-1 binds the NTD at the apex of the helical bundle, and inhibits the NTD-CTD interaction necessary for hexamer stabilization. Kelly et al. further characterized the binding of CAP1 to CA using X-ray crystallography and NMR, and observed that CAP1 binds into a hydrophobic pocket formed by the displacement of Phe32 (Kelly et al., 2007) (Figure 5B).

Other compounds that inhibit CA by a mechanism similar to CAP1 have been studied. Lemke et al. developed a high throughput assay for testing the effect of compounds on capsid formation and used it to screen a large library for CA-inhibitors. This led to the discovery of new inhibitors derived from Benzimidazole (BM) and benzodiazepine (BD) (Lemke et al., 2012) (Figure 5B). While both families bound CA at the same site (which is identical to the CAP-binding site), their modes of binding were different and had distinct effects on HIV-1 replication: while BD compounds inhibited capsid formation and release, BM compounds inhibited the formation of the mature, conical capsid after release from the cell. In later studies, the compounds were modified and improved - Tremblay et al. systematically modified the functional groups in a BM scaffold, yielding CA-inhibitors with IC_50_ values below 0.1 μM (Tremblay et al., 2012). Goudreau et al. developed BD-based compounds with IC_50_ values below 1 μM (Goudreau et al., 2013).

Recently, Goudreau et al. characterized a novel family of BM-based CA inhibitors that bind the NTD in a distinct site from CAP-1(Goudreau et al., 2013). One of the compounds induces the formation of an NTD-dimer with a non-native geometry (Lemke et al., 2013) (Figure 5C).

Several compounds that target the CA NTD were discovered by screening of compound libraries for inhibition of HIV replication in cells, with further studies revealing that CA was the target. Blair et al. and Shi et al. discovered the compound PF-74, which inhibits HIV replication in cells by binding to the NTD of CA and destabilizing the capsid structure (Blair et al., 2010; Shi et al., 2011). This causes premature uncoating of the virion during the replication cycle. On the other hand, Lamorte et al. discovered pyrrolopyrazolone based HIV inhibitors that bind CA at a similar binding site to PF74 and increase the stability of the viral capsid, thus interfering with the nuclear import of the stable synaptic complex (Lamorte et al., 2013) (Figure 5D). These results indicate that the viral replication cycle can be very sensitive to small changes in capsid stability in either direction, which further highlights the therapeutic potential of CA-binding molecules.

The CTD of CA is also a target for inhibition. Sticht et al. used phage display screening to identify peptides that bind CA and inhibit capsid formation. The authors discovered a peptide, termed capsid assembly inhibitor (CAI), that binds to the CTD and inhibits the formation of the mature capsid (Sticht et al., 2005). X-ray crystallography of the peptide-bound CTD (Ternois et al., 2005) showed that the peptide adopts a helical conformation and binds into a hydrophobic groove in the CTD, forming a five helix bundle. The binding of the peptide significantly alters the geometry of the dimerization interface (Figure 5E). Therefore, although the peptide does not directly bind the dimerization interface and does not destabilize the CTD dimer, it alters the geometry of the dimer interface, thus preventing the formation of the mature capsid. In a later study, the sequence of CAI was optimized using hydrocarbon stapling in order to stabilize the helical secondary structure of the peptide. This resulted in the peptide NYAD-1, which had improved cell penetration and activity *in vivo* (Zhang et al., 2008).

Bocanegra et al. used a rational design approach to inhibit capsid assembly. The authors synthesized a peptide, termed CAC1, derived from a helix in the dimerization interface of the CTD (Figure 5E), and also made several modifications in the peptide to increase its solubility and its affinity to the CTD. The peptides bound directly to the dimerization interface of CTD, inhibited capsid assembly and also had antiviral activity in cells (Bocanegra et al., 2011).

**Figure 6:**
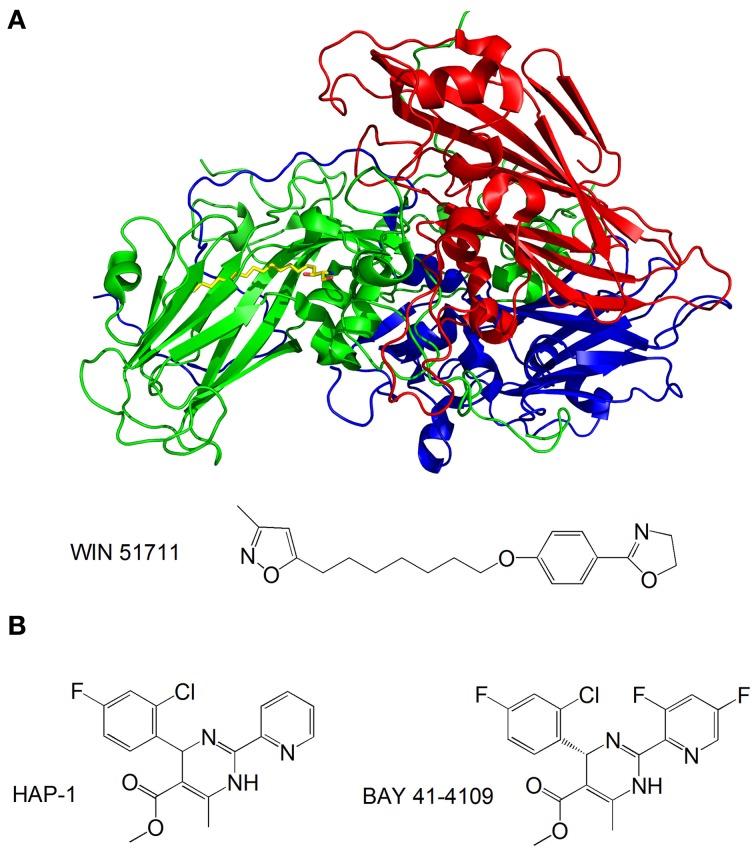
**(A)** Structure of the enterovirus 71 capsid proteins with WIN 51711. Three subunits of the capsid are in blue, green and red. WIN 51711 is in yellow. Based on Plevka et al. (2013); **(B)** Formulas of HBV capsid binding inhibitors (Stray et al., 2005; Stray and Zlotnick, 2006).

Capsid-binding molecules have been studied for other viruses. Plevka et al. used X-ray crystallography to study the effect of the capsid binding inhibitor WIN 51711 on the replication of enterovirus 71, which is associated with foot and mouth disease (Plevka et al., 2013). They discovered that WIN 51711 binds a pocket in one of the subunits involved in capsid formation and increases the stability of the capsid, thus restricting the dynamics of the capsid necessary for genome release and lowering the infectivity of the virus (Figure 6A).

The HBV is also being targeted by capsid-binding inhibitors. Heteroaryldihydropyrimidines (HAPs) are a class of compounds that inhibit HBV replication in tissue culture by interfering with capsid formation (Deres et al., 2003). The HBV capsid is made of 120 dimers of capsid protein (Cp) arranged in icosahedral symmetry (Bottcher et al., 1997). Stray et al. studied the effects of the two HAP compounds, HAP-1 and BAY 41-4109, on capsid formation *in vitro* (Stray et al., 2005; Stray and Zlotnick, 2006) (Figure 6B). While at substoichiometric concentrations the compounds increase the rate of capsid formation, at high concentrations they misdirect capsid formation and induce the formation of aberrant structures. The mechanism by which the compounds inhibit viral replication is still unclear—it is possible that the compounds do not antagonize HBV by directly inhibiting capsid formation, but rather disrupt the coordination of capsid assembly with other stages of the replication, or inhibit structural transitions necessary for the formation of mature, infectious capsids.

## Fibril-forming proteins

The equilibrium between monomers and fibrils plays a major role in many diseases, particularly neurodegenerative diseases such as Alzheimer's disease (Gilbert, 2013), Parkinson's disease and prion diseases (Salvatella, 2013). The inhibition of amyloid fibrillation is a major goal in drug development. Amyloid β fibrillation can be inhibited by short peptides derived directly from the sequences that mediate fibrillation (Tjernberg et al., 1996). Others methods involve rational design of inhibitors based on the structure of the interface between monomers (Sato et al., 2006; Sievers et al., 2011). This subject has been further reviewed elsewhere (Soto and Estrada, 2005; Belluti et al., 2013).

The monomer-fibril equilibrium can also be shifted toward the fibril, as has been shown in our laboratory for non-muscle myosin type II (NMII). NMII undergoes dynamic filament assembly and participates in cellular processes such as cell migration and cytokinesis. Ronen and Rosenberg et al. investigated the role of the non-helical C-terminal tailpiece in filament assembly (Ronen et al., 2010). They found that the tailpiece is intrinsically disordered and is divided into two oppositely charged regions. The positively charged part of the tailpiece interacts with an assembly incompetent fragment of NMII and induces its filamentation, while the negatively charged part affects the morphology of the filaments.

## Conclusion

Oligomerization plays a crucial role in the activity of many disease-related proteins and is therefore a promising target for therapeutic intervention. The molecules presented here were discovered by methods ranging from combinatorial screening methods such as phage display to rational, structure-based design. In most cases a combination of several methods was used. Many of the compounds were discovered in experiments that did not aim for modulating protein oligomerization, and their mechanism of action was elucidated later. However, as the knowledge of the oligomerization states and structure of the target proteins accumulates and methods that measure changes in the stability or structure of the oligomers become widespread, a growing number of active compounds are designed and screened for allosteric modulation of protein oligomerization. It is therefore not surprising that most of the compounds being developed target proteins with well characterized structures and oligomerization equilibria.

### The shiftides concept

**Figure 7:**
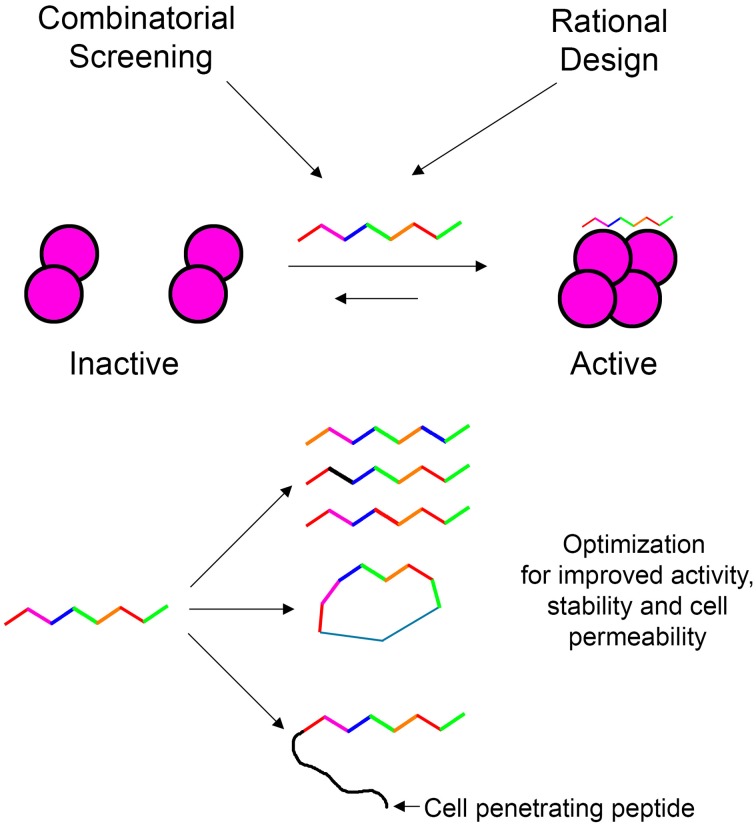
**Shiftides—peptides that modulate the oligomerization equilibrium of proteins**. Shiftides can be developed using combinatorial screening or rational design, and can be modified easily to improve their activity and pharmacological properties. An example of a protein in a dimer—tetramer equilibrium is given, but the principle can be applied to any oligomerization equilibrium.

In our laboratory we focus on peptides as tools for modulating protein oligomerization and we term them “shiftides.” These peptides bind specifically to a particular oligomeric state of the target protein and stabilize it. By doing so, these peptides shift the oligomerizaton equilibrium toward this specific oligomeric state. This way it is possible either to activate a protein by stabilizing an active oligomer or inhibit a protein by stabilizing an inactive oligomer. In this review, we have demonstrated the development of shiftides that target several proteins such as HIV-1 integrase (Hayouka et al., 2007; Maes et al., 2009), p53 (Gabizon et al., 2012) and non-muscle myosin IIC (Ronen et al., 2010). Shiftides can be discovered using rational design based on the sequences of proteins known to bind the target protein, using combinatorial approaches, or combining the two methods. The versatile chemistry of peptides enables the facile optimization of shiftide activity as well as improving pharmacological properties such as cell permeability (Wang et al., 2014) and proteolytic stability (Moretto et al., 2006; Dong et al., 2012) (Figure 7). Therefore, the shiftide approach can be applied effectively to a wide range of disease related proteins with dynamic oligomerizaion equilibria.

The number of disease-related oligomeric proteins that are being studied and characterized continues to grow (Lawrence et al., 2008; Ferré et al., 2010). A prominent example is G-protein coupled receptors (GPCRs). Many GPCRs are now known to form homo-and hetero-oligomers (Ferré et al., 2010), and the oligomerization of GPCRs can be critical to their activity (Jones et al., 1998) or significantly alter it (Azdad et al., 2008). The binding of agonists and antagonists can also induce conformational changes in the dimerization interface of GPCRs (Guo et al., 2005). Therefore, allosteric modulation of oligomerization may be a powerful approach for the development of drugs that target GPCRs in the future. As the number of potential targets grows, we expect that modulation of protein oligomerization will become a central therapeutic strategy for a variety of diseases.

### Conflict of interest statement

The authors declare that the research was conducted in the absence of any commercial or financial relationships that could be construed as a potential conflict of interest.
