# Supplementary material for: Allosteric modulation of protein oligomerization: an emerging approach to drug design
Source: Front Chem. 2014 Mar 24;2:9. doi: 10.3389/fchem.2014.00009 (PMC3982530; doi:10.3389/fchem.2014.00009)
Supplement: Supplementary file 1 [file DataSheet1.PDF]

Table S1: Target proteins and compounds reviewed in this study

Target protein	Target protein oligomerization equilibrium	Compound name	Activity	Reference
β-Tubulin	Monomer – fibril	Taxol	Inhibits microtubule assembly/disassembly dynamics	(Derry et al., 1995)
Phorphobilin ogen synthase	Octamer-hexamer via dimer	Morphlock-1	Stabilizes inactive hexamer allosterically	(Lawrence et al., 2008)
p53	Monomer – Dimer – Tetramer	Tetraguanidinium Ligand 1	Binds a patch of negatively charged residues on the surface of the p53 tetramerization domain	(Salvatella et al., 2004)
		Peptide CAN	Binds and increases the stability of the p53 tetramerization domain	(Martinell et al., 2006)
		Tetraguanidinium – calix[4]arene	Recovers the structure and tetramerization of the p53 R337H mutant	(Gordo et al., 2008, 2011)
		Imidazole – calix[6]arene	Increases the thermal stability and enhances the transcriptional activity of p53 R337H	(Kamada et al., 2010)
		p53CTD-binding peptides	The peptides bind specifically to p53 tetramers	(Gabizon et al., 2012)
AGR2	Monomer – Dimer (possibly higher oligomers)	AGR2 N-terminal peptide	Stabilizes higher oligomeric forms of AGR2	(Gray et al., 2013)
HIV-1 integrase (IN)	Dimer-Tetramer	Tetra-acetylated chicoric acid derivative	induces the formation of conformationally rigid IN tetramers that are unable to bind DNA in the necessary orientation for catalysis	(Kessl et al., 2009)
		IN-inhibiting shiftides	Inhibit IN and stabilize IN tetramers.	(Armon-Omer et al., 2008; Hayouka et al., 2010; Hayouka et al., 2007, 2008, 2010; Maes et al., 2009)

HIV-1 capsid protein (CA)	High order capsid structure composed primarily of hexamers bound via CTD dimers	CAP-1	Binds to the NTD and inhibits NTD-CTD interaction needed for hexamer formation	(Kelly et al., 2007; Tang et al., 2003)
		Benzimidazole CA inhibitors	Bind to the CAP-1 site and inhibit maturation of the capsid after release from the cell	(Lemke et al., 2012; Tremblay et al., 2012)
		Benzodiazepine CA inhibitors	Bind to the CAP-1 site and inhibit capsid formation and release	(Goudreau et al., 2013a; Lemke et al., 2012)
		Benzimidazole CA inhibitors with novel binding site	Bind to CA at a distinct site from CAP-1. One of the compounds induces the formation of a dimer with non-native geometry.	(Goudreau et al., 2013b; Lemke et al., 2013)
		PF-74	Binds to the NTD and causes premature uncoating during cell entry	(Shi et al., 2011)
		Pyrrolopyrazolone CA inhibitor	Binds the NTD and stabilizes the capsid, preventing nuclear import of stable synaptic complex	(Lamorte et al., 2013)
		Capsid Assembly Inhibitor (CAI) and NYAD-1	Bind the CTD and change the geometry of CTD dimers, inhibiting capsid formation	(Sticht et al., 2005; Ternois et al., 2005; Zhang et al., 2008)
		CAC1 Peptide	Bind the CTD dimerization interface and inhibit capsid formation	(Bocanegra et al., 2011)
Enterovirus 71 capsid	High order capsid structure	WIN 51711	Increases capsid stability and prevents genome release	(Plevka et al., 2013)
HBV capsid protein (Cp)	High order capsid structure	Heteroaryldihydro pyrimidines (HAPs)	At low concentrations increase the rate of capsid formation. At high concentrations induce formation of aberrant capsid structures	(Stray & Zlotnick, 2006; Stray et al., 2005)
Non Muscle Myosin II (NMIIC)	Dimer – filament	Non helical tailpiece of non muscle myosin II	The positively charged past of the tailpiece induces filamentation of the rod fragment of NMIIC, while the negatively charged part changes the morphology of the filaments	(Ronen et al., 2010, Rosenberg et al. 2013)
